# Oxytocin differentially modulates reward system responses to social and non-social incentives

**DOI:** 10.1007/s00213-024-06695-6

**Published:** 2024-10-04

**Authors:** Matthew D. Thurston, Lauren C. Ericksen, Maci M. Jacobson, Allison Bustamante, Vincent Koppelmans, Brian J. Mickey, Tiffany M. Love

**Affiliations:** 1 Centre for Advanced Imaging, The University of Queensland, Brisbane, QLD 4067 Australia; 2 Australian Institute for Bioengineering and Nanotechnology, The University of Queensland, Brisbane, QLD 4067 Australia; 3 Department of Psychiatry and Huntsman Mental Health Institute, The University of Utah, Salt Lake City, UT 84112 USA; 4 Department of Biomedical Engineering, The University of Utah, Salt Lake City, UT 84112 USA; 5 Department of Anesthesiology, The University of Utah, Salt Lake City, UT 84112 USA; 6 Interdepartmental Program in Neuroscience, The University of Utah, Salt Lake City, UT 84112 USA

**Keywords:** Oxytocin, Reward incentives, BOLD time-series, fMRI, Social cognition, Psychopharmacology

## Abstract

**Rationale:**

Oxytocin has been shown to modulate behavior related to processing of monetary incentives and to regulate social and reproductive behavior, yet little is known about how oxytocin differentially influences neural responses to social and non-social incentives.

**Objectives:**

We aimed to evaluate the effects of oxytocin administration on behavioral and neural responses to social and monetary incentives.

**Methods:**

Twenty-eight healthy adults (age 18–45 years) performed both monetary and social incentive tasks during blood oxygenation level dependent (BOLD) imaging. Intranasal oxytocin or placebo was administered before each scan using a double blind, randomized, cross-over design. Task performance and self-reported motivation and mood states were collected. Time-series analysis was conducted to assess the influence of oxytocin on the hemodynamic response in the ventral tegmental area and substantia nigra (VTA/SN) and nucleus accumbens (NAc).

**Results:**

Oxytocin demonstrated a multifaceted effect on VTA/SN and NAc when processing reward incentives, with it increasing BOLD response in VTA/SN and decreasing BOLD response in NAc during social incentive anticipation. A reversal of this was shown with decreased BOLD responses in the VTA/SN and increased BOLD response in the NAc during monetary incentive anticipation.

**Conclusions:**

Our findings suggest a more nuanced purpose of oxytocin when evaluating reward incentive decision making. It is possible that while oxytocin does increase salience to rewards, that it is more important for cognitive control when determining short-term versus long-term benefits in rewards. Future studies should more closely examine the relationship between oxytocin and delay discounting.

**Supplementary Information:**

The online version contains supplementary material available at 10.1007/s00213-024-06695-6.

## Oxytocin differentially modulates reward system responses to social and non-social incentives

Cognitive incentive processes help motivate an individual to pursue fulfillment again in the future and help to teach them efficient ways to attain their goals (Verharen et al. 2020). Dysfunctions in this system can lead to clinical issues related to addiction, depression, and other disorders (Poulton and Hester 2020). This system can be delineated neuroanatomically by the mesolimbic pathway, which encompasses reciprocal projections from the ventral tegmental area (VTA) and substantia nigra (SN) to the nucleus accumbens (NAc) (Mickey et al. 2016; Der-Avakian et al. 2016). Interactions of dopaminergic and oxytocin systems with the mesolimbic pathway have been associated with the cognitive processing of incentives (Bromberg-Martin et al. 2010; Mickey et al. 2016; Love 2014).

A recent meta-analysis attempted to determine whether the neuro circuitry of social versus non-social incentives were similar or different, by combining data from multiple studies that either used the monetary incentive delay task, used to measure non-social incentive activity, or a social incentive variant of the task known as the social incentive delay task (Gu et al. 2019). The study looked at activity specific to the anticipation period of these tasks across several regions related to reward. Overall, the study found almost identical activity during monetary and social incentive activity during win anticipation, supporting the hypothesis that social and monetary incentives are not processed differently. Another study also looked at the effects of oxytocin on monetary incentives in the VTA, substantia nigra, and NAc, and found that these regions were significantly modulated by oxytocin and that oxytocin effects were correlated with increased positive affect (Mickey et al. 2016).

Several other studies have examined the influences of oxytocin on social and non-social incentive processes. Hu et al. (2015) found that oxytocin particularly increased sensitivity to the anticipation of social feedback, and that this could possibly be due to increasing social salience in emotional and reward processing regions of the brain. Nawijn et al. (2016) showed that intranasal oxytocin could potentially reduce anhedonia related symptoms of reward processing in patients with PTSD, suggesting that intranasal oxytocin could also be useful for augmenting clinical psychotherapy sessions. Spetter et al. (2018) showed, in the case of food reward processing, oxytocin may simultaneously increase the reward response to food while also regulating the brain to select lower calorie foods, showing a possible multifaceted effect of oxytocin on reward processing. It should be noted that this multifaceted oxytocin effect was not replicated in monetary incentive reward processing. A study has shown that oxytocin may influence decision making accuracy (Zhou et al. 2024), while another study suggests that oxytocin does not influence reward network processing in males with or without autism spectrum disorder (Mayer et al. 2021).

Anticipatory neural models of reward incentives have shown that incentives are either approached or avoided through positively and negatively valenced states of arousal (Knutson and Greer 2008). These findings are supported by the theoretical basis of an affective circumplex modeling of mood states, based in valence and arousal (Watson et al. 1999). No study to date has directly examined oxytocin effects on the hemodynamic response in social and non-social incentive processing in the brain, and how these incentive processes are influenced by change in states of affective valence and arousal.

### The present study

In the present study we sought to directly compare oxytocin effects in the processing of social and non-social incentives in human adults using functional magnetic resonance imaging (fMRI). For our study we used two paradigms: one being the monetary incentive delay task (MID; Cooper and Knutson 2008), which has been demonstrated to measure non-social incentive processing in humans, and the social incentive delay task (SID), a social incentives version of the MID task, which we designed to directly compare non-social and social incentives in fMRI. When modeling avoid loss and win conditions over neutral, we hypothesized that the VTA, SN, and NAc would have greater peak BOLD response in the oxytocin group over the placebo group at around 6–8 s after social incentive anticipation (Hypothesis 1) and monetary incentive anticipation (Hypothesis 2) and that VTA, SN, and NAc would have a less pronounced BOLD undershoot at around 10–14 s after social incentive anticipation (Hypothesis 3) and monetary incentive anticipation (Hypothesis 4), in line with previous studies (Hu et al. 2015; Mickey et al. 2016; Nawijn et al. 2016). We further expected that these increases in BOLD would be correlated with positive affect and with motivation to pursue monetary and social incentives, within their respective tasks, in line with previous findings (Mickey et al. 2016; Hypothesis 5).

## Method

### Participants and design

Twenty-eight adults (8 females; 20 males) aged 18–45 (*M* = 29.78 *SD* ± 6.05) years performed the MID and SID tasks during fMRI scans. SID scans failed for 3 participants due to scanner technical difficulties. Excessive movement artifacts were found for 1 subject in both tasks, leaving a final within-subjects sample size of 27 MID and 24 SID. Each participant performed the tasks at two separate scanning sessions, once when given an intranasal oxytocin intervention, and once when given a matched intranasal placebo intervention. The order of the tasks and intervention sessions were both counterbalanced. Exclusionary criteria included any form of MRI exclusion criteria such as metal implants or pregnancy, and any current or past psychiatric diagnosis, determined via the Mini International Neuropsychiatric Interview (Sheehan et al. 1998). All data was collected at the University of Utah. Informed consent was collected for all subjects. The study was reviewed and approved by the University of Utah Institutional Review Board. Protocols were registered on ClinicalTrials. gov (identifier: NCT02652195). Due to an oxytocin manufacturing change, the trial was closed earlier than expected. This study covers a sub-analysis of healthy controls to better understand the characterization of our findings in a healthy population, prior to exploring the effects in patients with alcohol use disorder.

### Oxytocin administration

**Fig. 1:**
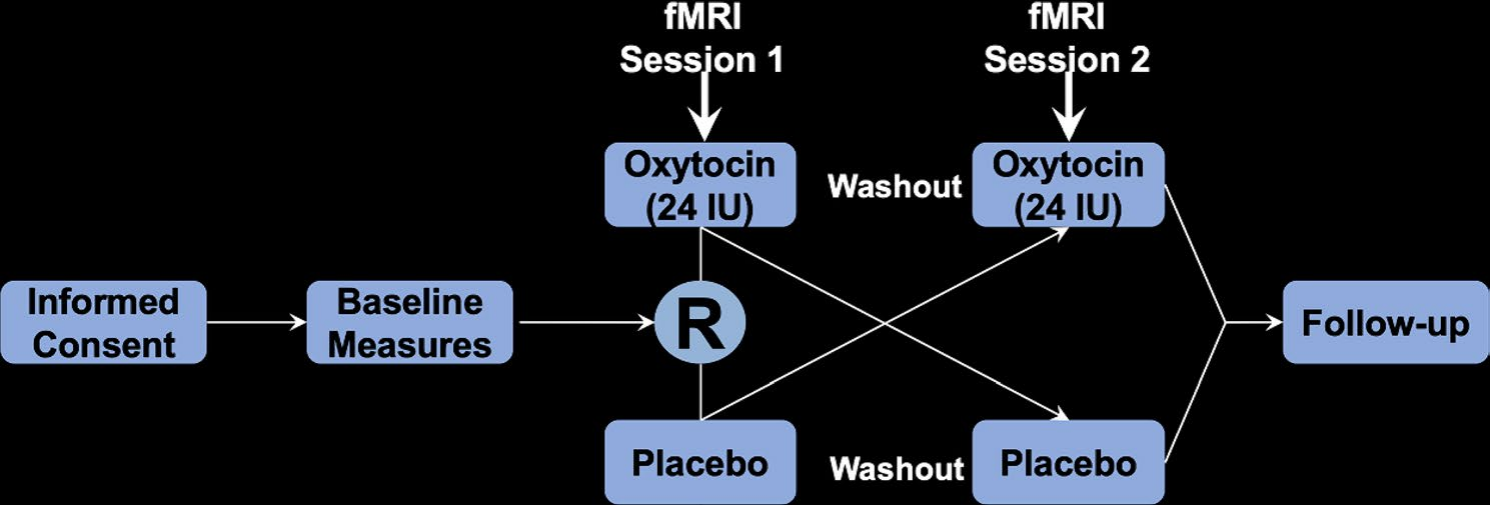
Randomized order of allocation for placebo versus oxytocin trials

Oxytocin and matched placebo solutions were obtained from PharmaWorld (Zurich, Switzerland) and stored in a continuously temperature-monitored 4 °C refrigerator. At the scanning session, oxytocin or placebo was self-administered intranasally following verbal instructions and nasal delivery procedure was demonstrated by study staff. Participants and research staff involved in the administration remained blinded to allocation (oxytocin versus placebo). The order of allocation was determined with a computer-generated random allocation sequence without blocking (SAS software, Cary, NC, see Fig. 1). All allocations and drug assignments were concealed to both participants and study staff, except for research pharmacy personnel who prepared the drugs and did not interact with the participants. Intranasal oxytocin was administered approximately 45 min prior to scanning sessions.

## Behavioral measures

We collected measures of positive and negative mood states using the Positive and Negative Affect Schedule (PANAS; Watson et al. 1988). We also collected measures via visual analog scales (VAS) on five emotions associated with a participant’s perception of social and monetary incentives, for a total of ten items (social or monetary; avoid loss, win, or neutral; five emotions each). The five emotions we measured associated with their perception of social and monetary incentives were: happiness, excitement, unhappiness, fearfulness, and motivation. The social VAS asked participants to rate “.. how you felt about each cue during the task where you had the chance to play on someone else’s behalf” and the monetary VAS asked participants to rate “.. how you felt about each cue during the task where you had the chance to win money for yourself.” The five emotions were prompted as “How happy?”, “How excited?”, “How unhappy?”, “How fearful?”, “How motivated?”. The electronic VAS scales were completed by selecting a slider with “Not at all” on the left end of the scale and “Extremely” on the right end of the scale (0-100 was the quantitative). Some participants did not complete all behavioral measures to stay within the time-sensitivity of oxytocin administration and the fMRI paradigms. A total of 20 participants completed the PANAS and 19 participants completed the VAS measures throughout sampling.

### Paradigms

#### The monetary incentive delay task

We used a slightly modified version of the MID task (Knutson et al. 2001; Cooper and Knutson 2008) to measure fMRI associated with non-social incentives. The task included 5 conditions: large win, small win, large avoid loss, small avoid loss, and neutral. Each trial started with a condition cue to indicate what condition the trial would be. The cue (500ms) was followed by a brief fixation mark (2–6 s) and then a target star (2–6 s); the subject was instructed to click on a button box before the star disappeared. If successful, they received feedback (500ms) stating that they succeeded and how much money they either gained or avoided losing. If they failed to hit the target, they received feedback that they failed along with the amount of money they either lost or failed to gain. Trial difficulty was researcher controlled to ensure roughly 66% accuracy, to sustain sufficient win versus loss power for analysis. We further tested accuracy and reaction times between placebo and oxytocin to confirm accuracy rates and measure differences in reaction times.

#### The social incentive delay task

**Fig. 2:**
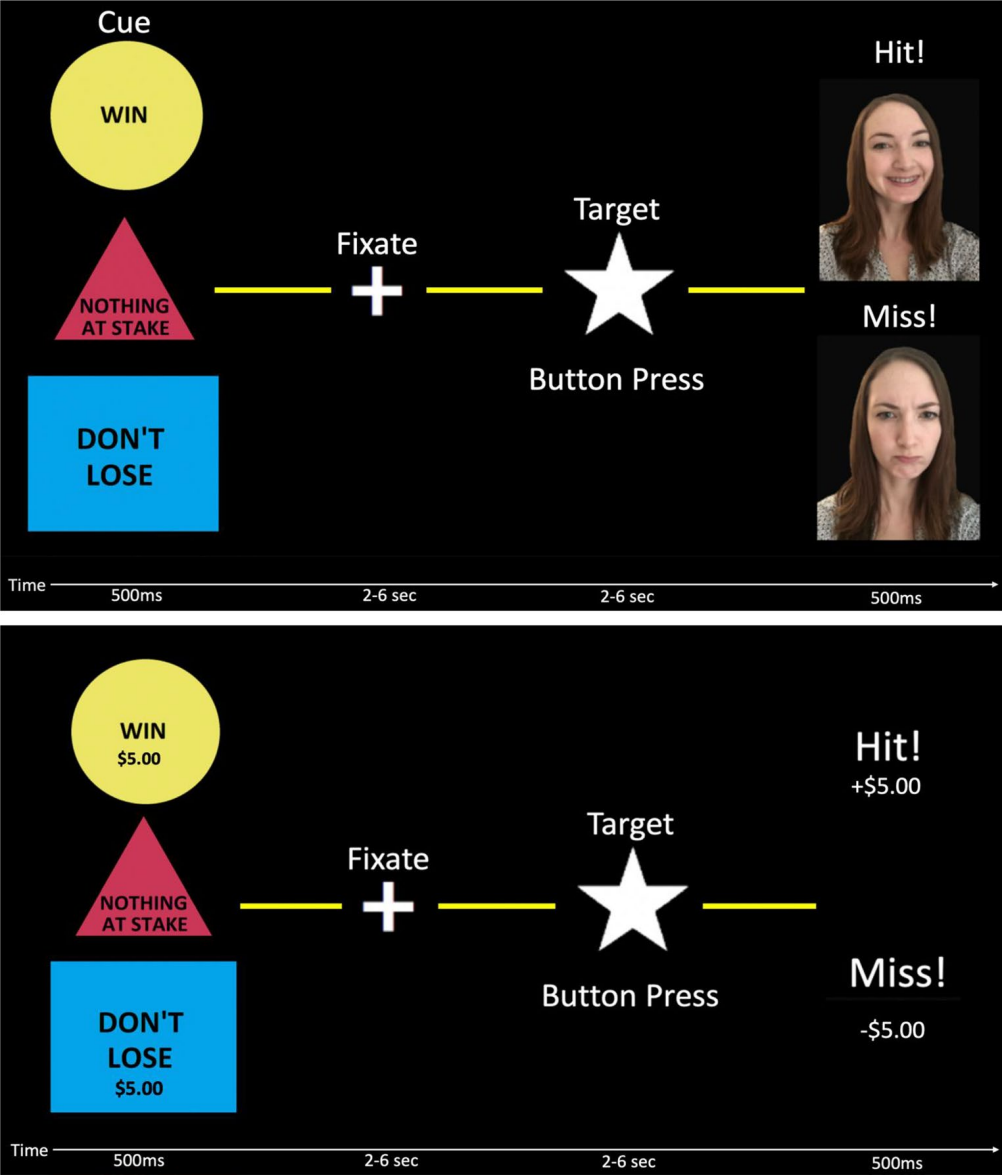
Experimental design of the SID and MID. Top image: SID task; Bottom image: MID task

The SID task was designed similarly to the MID task above, however it was modeled to elicit social reward rather than monetary reward. There were no small or large versions of win or avoid loss. Participants met with a partner at their lab visit before the scan. The participants were told that they would be winning money for their partner, which they would then be donating to a charity. Participants were given time to get to know and connect with the partner. Participants were told that their scan and results on the SID would be monitored by the partner. Once in the scanner, participants were shown a picture of the partner smiling with the word “Hit!” for success feedback and a picture of the partner frowning with the word “Miss!” for failure feedback, or a pixelated face for control. All remaining segments of the task were congruent to the MID (see Fig. 2 for paradigm design). Trial accuracy was again research controlled to a rate of 66% and we tested placebo versus oxytocin effects of accuracy and reaction times.

### Power analysis

Prior to onset of data collection, we conducted an fMRI-based power analysis using the fMRIpower software package (fmripower.org; Mumford and Nichols 2008). While our power analysis recommended a sample size of 45 to achieve a 90% threshold of power to detect an effect, due to the change in oxytocin manufacturing this study was cut short, and thus our sample has fallen short of 45. We believe our current sample still provides effects of interest for the literature.

### fMRI acquisition

Whole brain functional imaging was performed on a Siemens Prisma Fit 3-T system using a T2*-sensitive simultaneous multi-slice echo planar imaging sequence (TR = 0.8s, TE = 30 ms, flip angle = 52^°^). All data was preprocessed in fmriprep (Esteban et al. 2018) and included regressors for cerebral spinal fluid, white matter, and motion. Anatomy was imaged using a T1-weighted magnetization-prepared rapid gradient-echo (MPRAGE) sequence (TR = 2.5s, TE = 2.9 ms, flip angle = 8^°^).

### fMRI analysis

**Fig. 3:**
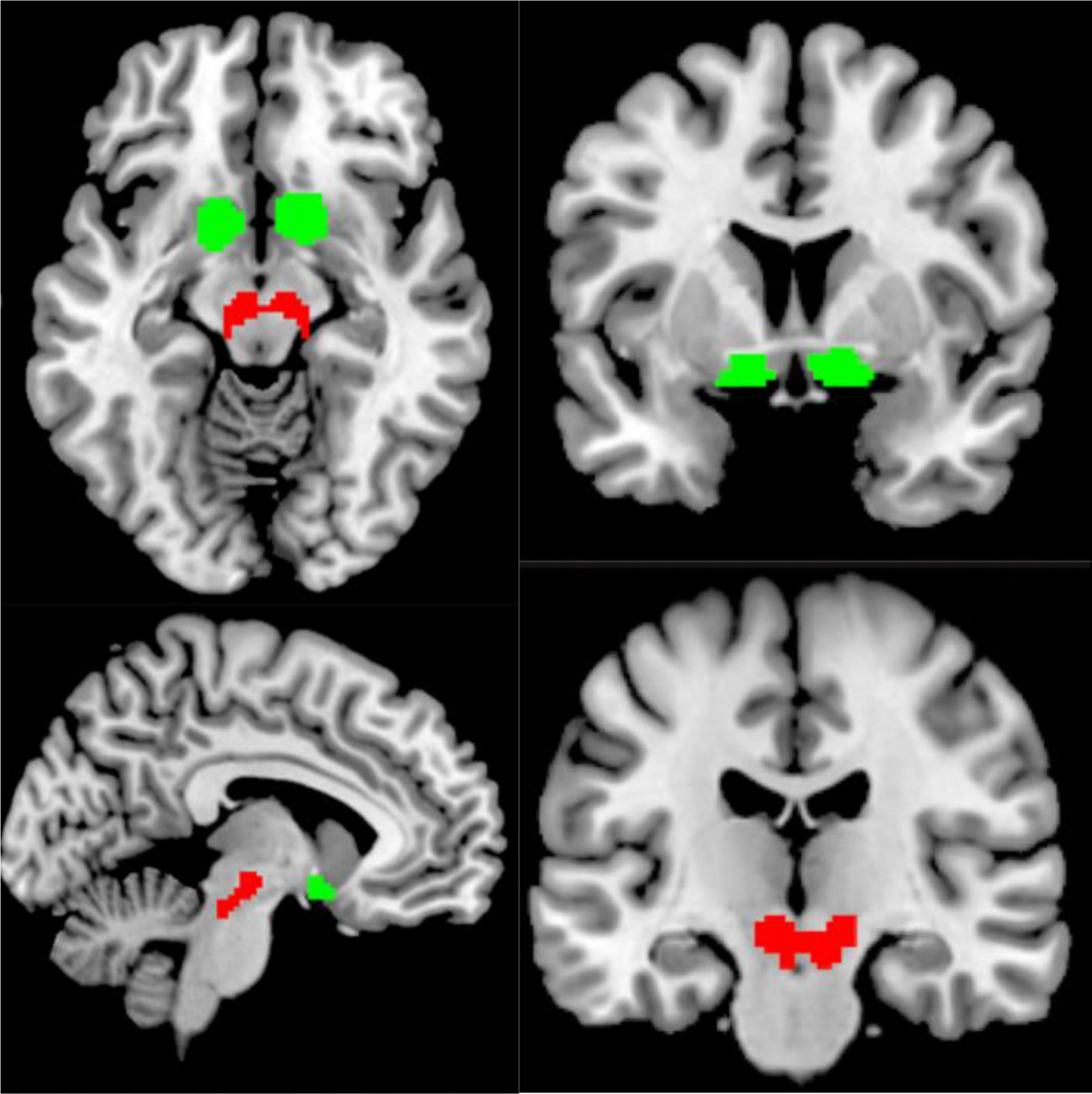
Masks used for region of interest analysis; contains the VTA/SN (red), and NAc (green)

Based on our previous findings that oxytocin changes the shape of the hemodynamic response function in the mesolimbic pathway (Mickey et al. 2016), we extracted time-series from each region of interest using MarsBaR in SPM 12 (Brett et al. 2002): bilateral VTA/SN (merged as one ROI), and bilateral NAc (see Fig. 3 for ROIs). The NAc was defined using the individual brain atlas process in SPM12 (IBASPM) and the VTA/SN was defined using the ROI used in Mickey et al. (2016). Each time-series consisted of beta-weights from condition regressors of the tasks, averaged into ten 2-second time bins (e.g. time bin 1 = the average beta-weight recording across 0 to 2 s post cue onset), starting from the onset of the condition cue to 20 s post onset. To compare the SID and MID tasks more easily, the small win and loss conditions of the MID were excluded in the main analysis. The remaining MID condition regressors included the following: large win anticipation, large avoid loss anticipation, and neutral anticipation. The SID condition regressors included the following: win anticipation, avoid loss anticipation, and neutral anticipation. To examine oxytocin effects across time for the entire tasks, all conditions (win, loss, neutral) were averaged together into a single time-series for placebo and a single time-series for oxytocin.

### Time-series analysis

We used linear mixed modeling in R software (version 4.0.3) using the nlme package (version 3.1). Regressor beta weights were modeled for each paradigm separately (MID & SID). Fixed effects were time, drug (oxytocin versus placebo), condition (win, loss, or neutral), region of interest (ROI), and all interactions; the random effect was subject. Type-II Wald chi-square tests were used for hypothesis testing. The linear effect of time was modeled from 6 to 14 s post onset of anticipation cue, where we saw the most variability of interest in the hemodynamic response. We also correlated differences between oxytocin and placebo at each time bin for each region of interest, with the PANAS and all VAS social and monetary measures. ANOVAs were conducted to determine oxytocin effects on task performance, PANAS, and VAS measures. Neural-behavioral correlates with Bonferroni correction for all PANAS and VAS behavioral measures (p-threshold = 0.007) were reported.

## Results

### Behavior

#### MID task performance

We found no effects of oxytocin on MID accuracy for win (*t*(49) = 0.32, *p* = 0.75; oxytocin: *M* = 0.67 *SD* ± 0.04; placebo: *M* = 0.67 *SD* ± 0.03), avoid loss (*t*(49) = 1.01, *p* = 0.32; oxytocin: *M* = 0.70 *SD* ± 0.01; placebo: *M* = 0.69 *SD* ± 0.02), or neutral (*t*(49) = 0.56, *p* = 0.57; oxytocin: *M* = 0.63 *SD* ± 0.19; placebo: *M* = 0.61 *SD* ± 0.18). We also found no effects of oxytocin on MID reaction time for win (*t*(49) = 0.76, *p* = 0.45; oxytocin: *M* = 0.19 *SD* ± 0.01; placebo: *M* = 0.20 *SD* ± 0.01), avoid loss (*t*(49) = 0.70, *p* = 0.48; oxytocin: *M* = 0.13 *SD* ± 0.02; placebo: *M* = 0.13 *SD* ± 0.02), or neutral (*t*(49) = 0.28, *p* = 0.78; oxytocin: *M* = 0.20 *SD* ± 0.01; placebo: *M* = 0.20 *SD* ± 0.01).

#### SID task performance

We found no effects of oxytocin on SID accuracy for win (*t*(49) = 0.35, *p* = 0.73; oxytocin: *M* = 0.65 *SD* ± 0.04; placebo: *M* = 0.65 *SD* ± 0.04), avoid loss (*t*(49) = 0.21, *p* = 0.84; oxytocin: *M* = 0.65 *SD* ± 0.03; placebo: *M* = 0.66 *SD* ± 0.06), or neutral (*t*(49) = 0.36, *p* = 0.72; oxytocin: *M* = 0.67 *SD* ± 0.19; placebo: *M* = 0.65 *SD* ± 0.18). We also found no effects of oxytocin on SID reaction time for win (*t*(49) = 0.34, *p* = 0.73; oxytocin: *M* = 0.20 *SD* ± 0.02; placebo: *M* = 0.20 *SD* ± 0.01), avoid loss (*t*(49) = 0.08, *p* = 0.94; oxytocin: *M* = 0.17 *SD* ± 0.01; placebo: *M* = 0.17 *SD* ± 0.01), or neutral (*t*(49) = 0.23, *p* = 0.82; oxytocin: *M* = 0.21 *SD* ± 0.02; placebo: *M* = 0.20 *SD* ± 0.01).

#### Mood states related to monetary incentives

We found no effects of oxytocin related to self-perceived happiness across feedback conditions (*F*(1, 52) = 0.07, *p* = 0.79), excited (*F*(1, 53) = 0.15, *p* = 0.70), unhappiness (*F*(1, 53) = 0.15, *p* = 0.70), fearfulness (*F*(1, 53) = 0.01, *p* = 0.95), nor motivation (*F*(1, 53) = 0.06, *p* = 0.81) in relation to monetary incentives.

#### Mood states related to social incentives

We found no effects of oxytocin on self-reported happiness (*F*(1, 52) = 0.35, *p* = 0.56), excited (*F*(1, 52) = 1.37, *p* = 0.25), unhappiness (*F*(1, 52) = 0.001, *p* = 0.97), fearfulness (*F*(1, 52) = 0.03, *p* = 0.87), or motivation (*F*(1, 52) = 0.06, *p* = 0.80) in relation to social incentives.

#### Oxytocin effects on affective state (PANAS)

We found no effects of oxytocin on positive or negative affect (*F*(1, 53) = 2.16, *p* = 0.15).

### Neural responses during the tasks

See Table 1 for complete linear mixed model of the MID and Table 2 for the complete linear mixed model of the SID.

#### Main effects of time

**Fig. 4:**
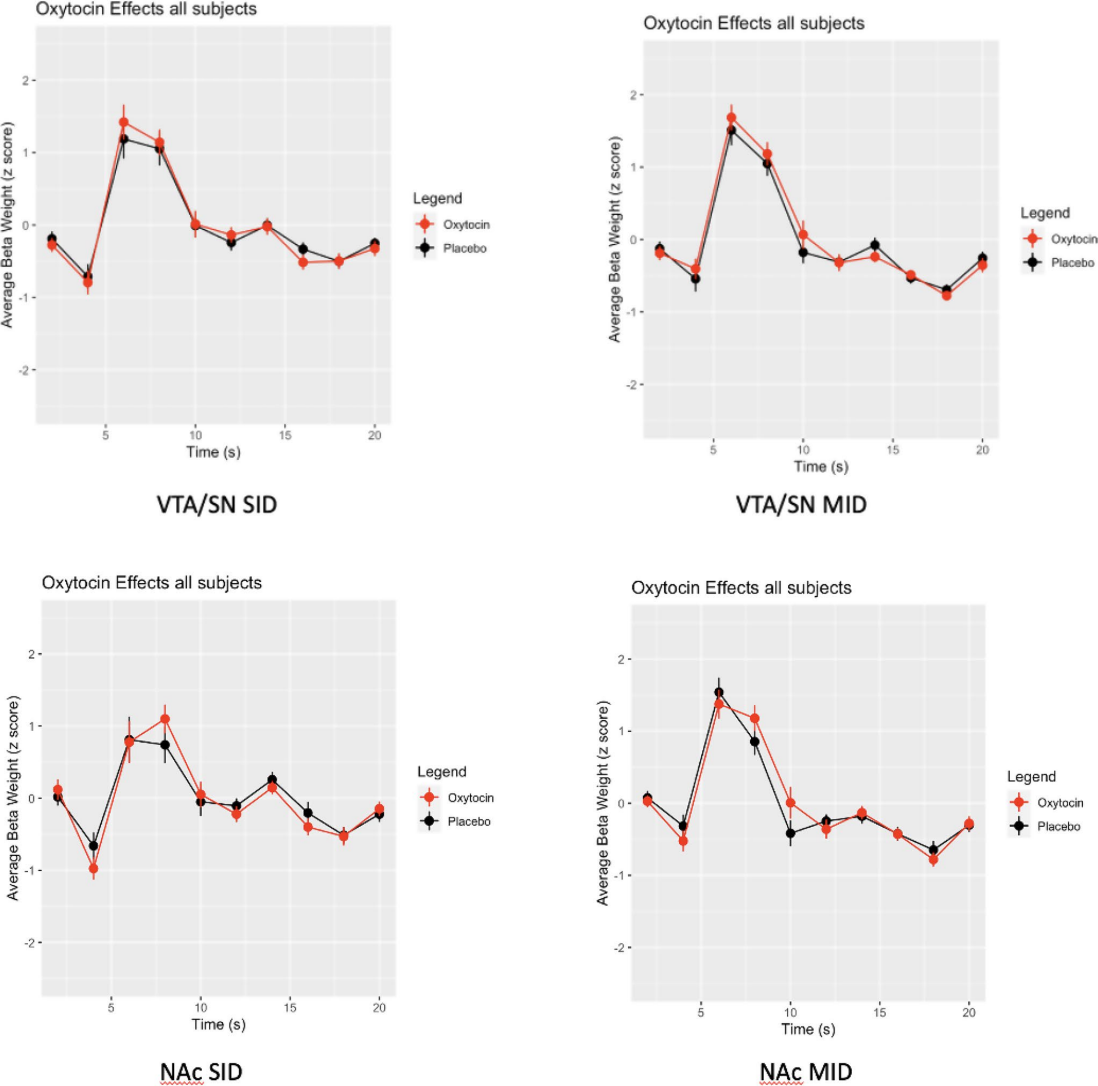
Time-series of oxytocin effects across all conditions

**Fig. 5:**
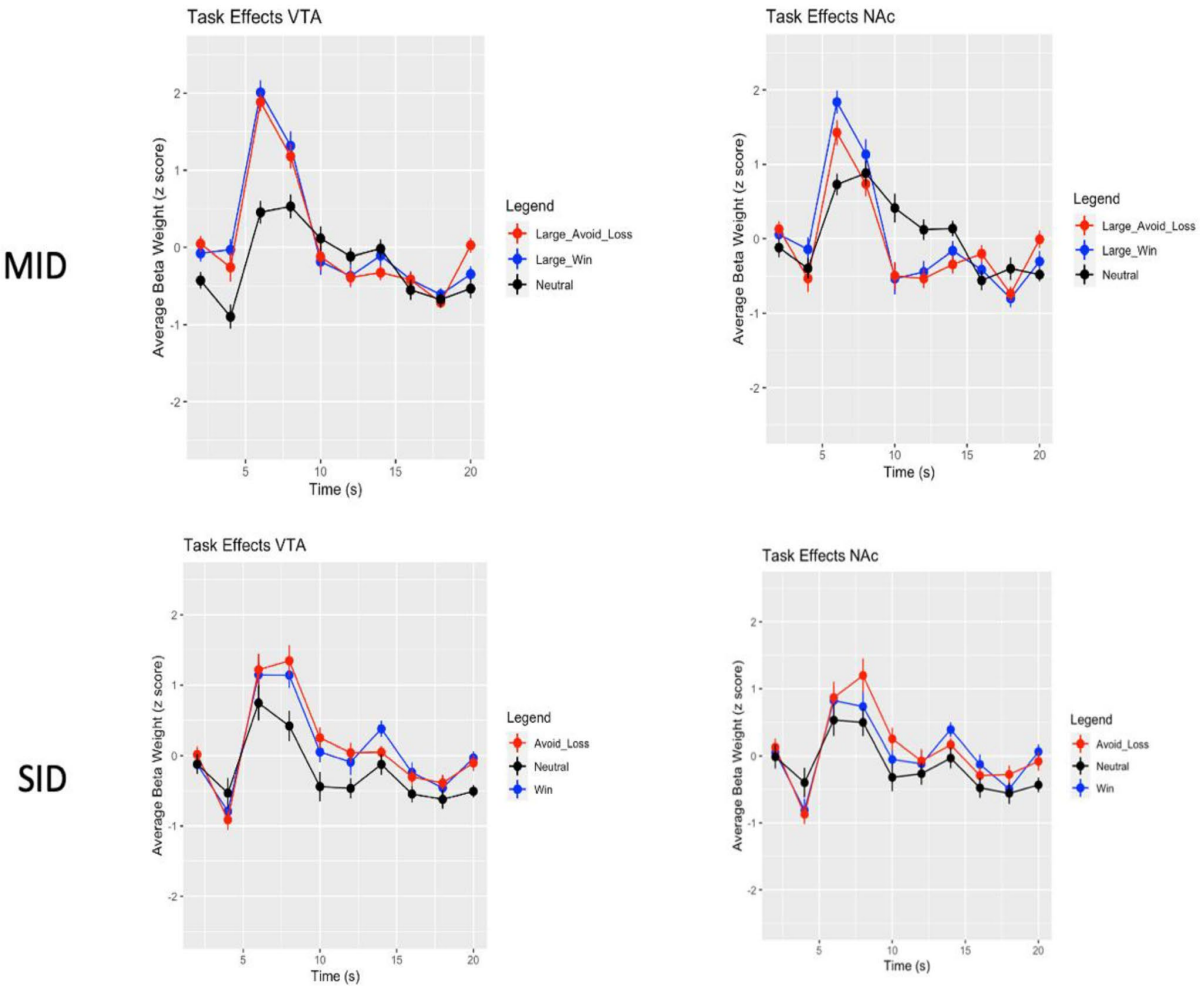
Response versus condition, Collapsed across oxytocin and placebo

For both the MID (*χ*^*2*^(3) = 276.74, *p* < 0.0001) and SID (*χ*^*2*^(3) = 146.56, *p* < 0.0001) we found an effect of time, with both tasks eliciting the expected peak BOLD responses at 6–8 s in both ROIs (see Figs. 4 and 5).

**Table 1:** Linear mixed model of hemodynamic response in ROIs with oxytocin versus placebo: Monetary incentive delay task

	χ^2^	df	*p*
Factor			
Time	276.74	3	< 0.0001*
Oxytocin	1.84	1	0.18
Condition	13.79	2	0.001*
ROI	0.81	1	0.37
Time X Oxytocin	5.81	3	0.12
Time X Condition	37.14	6	< 0.0001*
Time X ROI	1.72	3	0.63
Oxytocin X Condition	7.53	2	0.02*
Oxytocin X ROI	0.63	1	0.43
Condition X ROI	12.18	2	0.002*
Time X Oxytocin X Condition	1.19	6	0.98
Time X Oxytocin X ROI	0.74	3	0.86
Time X Condition X ROI	3.43	6	0.75
Oxytocin X Condition X ROI	7.24	2	0.03*
Time X Oxytocin X Condition X ROI	1.93	6	0.93

**Table 2:** Linear mixed model of hemodynamic response in ROIs with oxytocin versus placebo: social incentive delay task

	χ^2^	df	*p*
Factor			
Time	146.56	3	< 0.0001*
Oxytocin	0.49	1	0.48
Condition	43.39	2	< 0.0001*
ROI	0.005	1	0.94
Time X Oxytocin	1.49	3	0.68
Time X Condition	11.17	6	0.08
Time X ROI	5.31	3	0.15
Oxytocin X Condition	6.64	2	0.04*
Oxytocin X ROI	0.33	1	0.56
Condition X ROI	6.08	2	0.048*
Time X Oxytocin X Condition	6.80	6	0.34
Time X Oxytocin X ROI	1.57	3	0.67
Time X Condition X ROI	4.83	6	0.57
Oxytocin X Condition X ROI	2.92	2	0.23
Time X Oxytocin X Condition X ROI	2.67	6	0.85

#### Responses vary across condition and brain region

For the MID we found a two-way interaction effect of time X condition, *χ*^*2*^(6) = 37.14, *p* < 0.0001 and a main effect of condition, *χ*^*2*^(2) = 13.79, *p* = 0.001. For the SID we found a main effect of condition, *χ*^*2*^(2) = 43.39, *p* < 0.0001, with both tasks in general eliciting greater responses during avoid loss and win trials, over neutral trials (see Fig. 5). We found a condition X ROI interaction for both the MID (*X*^*2*^(2) = 12.18, *p* = 0.002) and the SID (*χ*^*2*^(2) = 6.08, *p* = 0.048). Figure 5 illustrates this variation between ROIs and across condition for each task.

### Effects of oxytocin on neural responses

#### Oxytocin does not affect response amplitude or shape when collapsing across condition

We saw no main effects of oxytocin on hemodynamic response in either the MID, *X*^*2*^(1) = 1.84, *p* = 0.18, or the SID, *X*^*2*^(1) = 0.49, *p* = 0.48. We also saw no oxytocin X time interactions in either the MID, *X*^*2*^(3) = 5.81, *p* = 0.12, or the SID, *X*^*2*^(3) = 1.49, *p* = 0.68, indicating the amplitude and shape of BOLD response remains constant across oxytocin and tasks (see Fig. 4).

#### Oxytocin modulates condition-specific responses

**Fig. 6:**
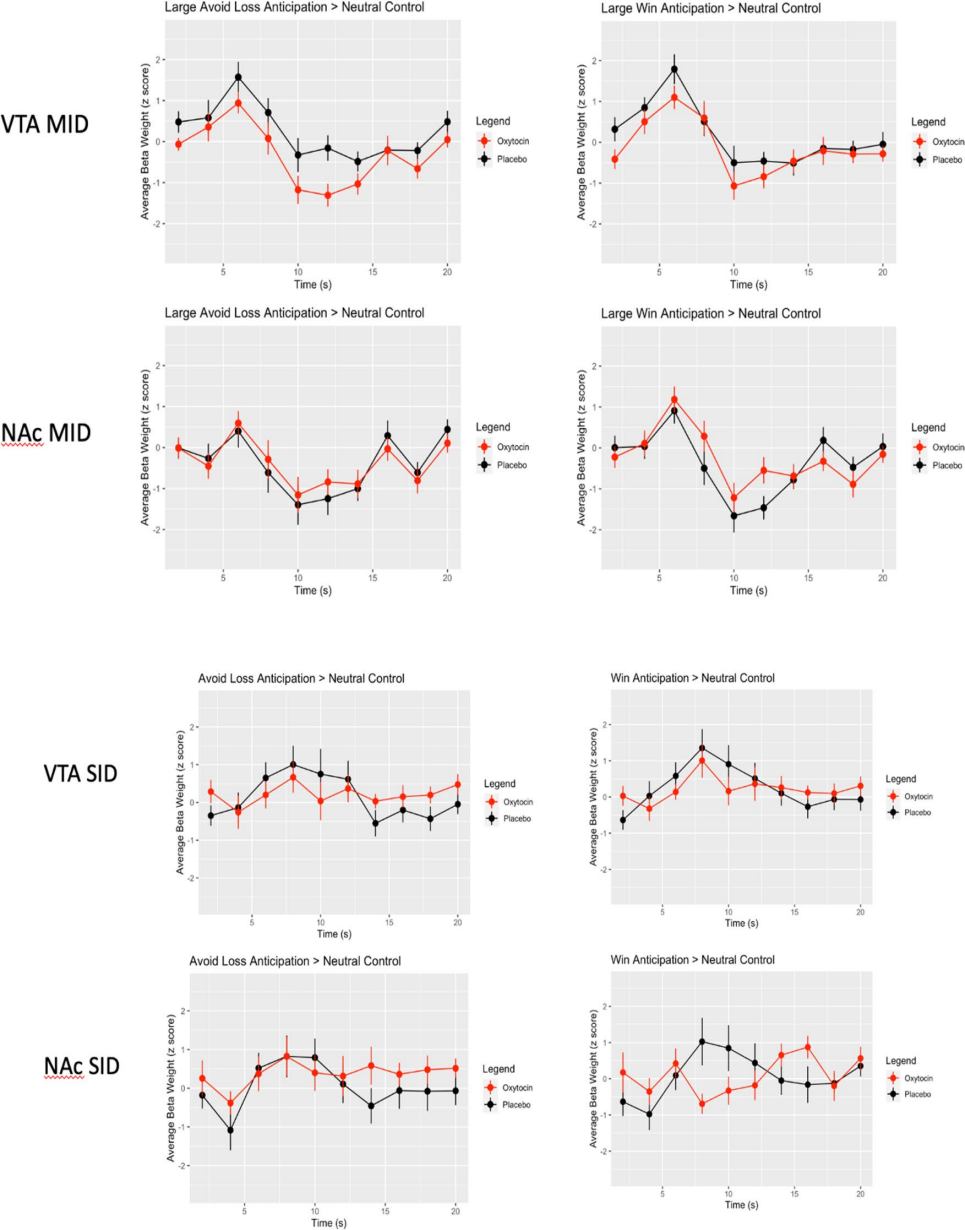
Time-series of oxytocin effects by condition after subtraction of neutral control

For both the MID (*χ*^*2*^(2) = 7.53, *p* = 0.02) and the SID (*χ*^*2*^(2) = 6.64, *p* = 0.04), we saw an oxytocin X condition interaction, showing that oxytocin modulates condition-specific responses during both monetary and social incentive processing (see Fig. 6). For the MID we also found a three-way interaction effect of oxytocin X condition X ROI, *χ*^*2*^(2) = 7.24, *p* = 0.03, suggesting an ROI-specific effect of oxytocin only during the MID (see Fig. 6).

MID contrasts with the neutral condition subtracted (Fig. 6) indicated that oxytocin tended to reduce BOLD signal in VTA/SN and increase BOLD signal in NAc. The largest effect of oxytocin was an enhancement of the BOLD undershoot at 12 s in the VTA/SN during the monetary loss condition. With the SID task, contrasts with the neutral condition subtracted (Fig. 6) showed that oxytocin tended to flatten the BOLD response across conditions in both VTA/SN and NAc. The most prominent effect was an oxytocin-induced suppression of the BOLD signal peak at 8 s in the NAc during the social win condition.

### Neural-behavioral correlations

For the VTA/SN during the MID, increases in feelings of happiness for monetary reward were correlated with decreases in BOLD response from oxytocin (oxytocin – placebo) at 10–12 s post-onset, *r*(17) = -0.61, *pcorr* < 0.007. For the NAc during the SID, increases in feelings of motivation for social reward were correlated with increases in BOLD response from oxytocin (oxytocin – placebo) at 8–10 s post-onset, *r*(17) = 0.64, *p*corr < 0.007.

All other correlations of BOLD response and task-related mood states or PANAS scales across our 6–14 post-onset time window of interest were not statistically significant (*pcorr* > 0.007). For significant uncorrected neural-behavioral correlates, see Supplemental Table 1.

## Discussion

Contrary to Hypothesis 1 we found no significant increase in peak BOLD response (6–8 s) in the oxytocin group during SID anticipation, across both ROIs and both conditions over neutral. In further contradiction to Hypothesis 1, we saw a decrease in BOLD peak in the NAc during win anticipation specifically. Contrary to Hypothesis 2 we found no significant increase in peak BOLD response (6–8 s) in the oxytocin group during MID anticipation, across both ROIs and both conditions. In further contradiction to Hypothesis 2, we observed a decrease in peak BOLD response in the oxytocin group during MID anticipation, specifically in the VTA/SN during win anticipation over neutral of the MID. Congruent with our Hypothesis 3, we found a less pronounced BOLD undershoot at 14 s in both the VTA/SN and the NAc, during SID avoid loss over neutral trials specifically. In contradiction to Hypothesis 3, we found a more pronounced BOLD undershoot at 10 s during SID win anticipation over neutral. Congruent with our Hypothesis 4, we found a less pronounced BOLD undershoot at 12 s during win anticipation of the MID, however in contradiction to our Hypothesis 4, we found a more pronounced BOLD undershoot from 10 to 12 s of the MID. Contradictory with our Hypothesis 5, feelings of happiness for monetary reward were associated with less BOLD response at 10–12 s post MID anticipation in the VTA/SN, however congruent with our Hypothesis 5, feelings of motivation for social reward were associated with increases in BOLD response at 8–10 s post SID anticipation in the NAc. Several uncorrected neural correlate effects were observed but these may be due to smaller effect sizes or false positive findings. Further, our lack of whole-brain analysis is related to the fact that the factor of time is collapsed and we wanted to underscore the importance of measuring oxytocin effects in BOLD via time-series analyses.

Our SID BOLD findings for oxytocin suggests that oxytocin may function in a similar way to social reward as it was shown to function for food reward in Spetter et al. (2018). The less pronounced undershoot of the BOLD response in VTA/SN and NAc, may enhance the salience of social rewards, while the reduction of the peak BOLD response in the NAc may simultaneously be regulating a selective filter about what social rewards to actively engage in. We saw an opposite pattern in the MID where oxytocin may be decreasing reward salience to monetary incentives in the VTA/SN while at the same time increasing the NAc motivation to interact with monetary rewards. Those motivated for social reward may experience less of a reward filter from oxytocin, while those who associate happiness with monetary reward may have a reduced reward salience to monetary incentives.

Like Spetter et al. (2018), these findings suggest a more multifaceted influence of oxytocin on reward incentive processing. Specifically, that oxytocin influences both the salience of reward and the ability to engage in cognitive control in relation to reward. These findings would be further congruent with other previous studies where Hu et al. (2015) demonstrated an increase in sensitivity to social feedback from oxytocin, Nawijn et al. (2016) showed oxytocin could increase reward motivation for patients with PTSD, and that an increase in cognitive control may be linked to more decision-making accuracy, as shown in Zhou et al. (2024). The contradictive findings in Mayer et al. (2021) may be due to the emphasis of looking at oxytocin via affective circuits rather than cognitive control circuitry.

Together these findings and literature may suggest that oxytocin isn’t solely important for improving the salience to reward processing but that it is more pertinent to the selectivity and cognitive control associated with reward engagement. In essence, that oxytocin may improve common cognitive processes such as delay discounting, which allow an individual to pursue an important reward over a long period of time without receiving any benefits from it until the necessary tasks associated with the long-term pursuit are completed. This would be associated with the reduction in the NAc activity that allows a person to be more effective at restraining themselves from engaging in short-term rewards that may disrupt their ability to pursue a long-term reward, while oxytocin also seems to stimulate the salience to that long-term reward over said continuous pursuit. A disruption in the oxytocin mechanisms might explain why in instances of substance abuse, patients with substance abuse disorders often still desire to pursue social and non-social rewards, yet they are unable to sustain the cognitive control necessary to not disrupt the natural process of attaining long-term rewards associated with such pursuits.

Limitations of our study included that our sample was heavily sex biased towards males but the within-subject power elicited by both fMRI tasks may have compensated for this a bit. Our decision to also exclude small win and avoid loss trials from the MID may have reduced the power of our MID data but with previous findings showing a difference in effect size between small and large MID rewards (Mickey et al. 2016), we felt that this may introduce too much complexity into our models. This study was also further limited by the experimental environment itself not fully simulating real-world reward and social encounters in a more natural setting. Our study also fell short of our target sampling of 45, due to the manufacturing change in our oxytocin supplier. Future studies may wish to look at oxytocin effects between sex, as well as between healthy controls and psychiatric populations known to having reward incentive processing deficits, such as in substance and alcohol abuse disorders, in a way that can better measure whether oxytocin is influencing their deficits in delay-discounting and/or cognitive control specifically.

## Electronic supplementary material

Below is the link to the electronic supplementary material.

Supplementary Material 1
