# Supplementary material for: Oxytocin differentially modulates reward system responses to social and non-social incentives
Source: Psychopharmacology (Berl). 2024 Oct 4;242(3):449–60. doi: 10.1007/s00213-024-06695-6 (PMC11861123; doi:10.1007/s00213-024-06695-6)
Supplement: Supplementary file 1 — Supplementary Material 1 [file 213_2024_6695_MOESM1_ESM.docx]

**Supplementary Table 1**

*Uncorrected Findings of Neural-Behavioral Correlates*

| Correlate 1 | Correlate 2 | *r* | *df* | *p* |
| --- | --- | --- | --- | --- |
| **NAc During MID** | | | | |
| Motivation for Monetary Reward | 6-8 seconds post onset MID anticipation | 0.52 | 17 | 0.02 |
| **VTA/SN During MID** | | | | |
| Excitedness for Monetary Reward | 10-12 seconds post onset MID anticipation | -0.48 | 17 | 0.04 |
| Positive Affect | 12-14 seconds post onset MID anticipation | -0.45 | 18 | 0.022 |
| **NAc During SID** | | | | |
| Happiness for Social Reward | 6-8 seconds post onset SID anticipation | 0.48 | 17 | 0.04 |
| Motivation for Social Reward | 6-8 seconds post onset SID anticipation | 0.57 | 17 | 0.01 |
| **VTA/SN During SID** | | | | |
| Excitedness for Social Reward | 10-12 seconds post onset SID anticipation | 0.50 | 17 | 0.04 |
| Positive Affect | 10-12 seconds post onset SID anticipation | 0.46 | 18 | 0.03 |
| Positive Affect | 12-14 seconds post onset SID anticipation | -0.50 | 17 | 0.02 |
